# Knowledge and Perception of Pharmacy Students toward Telepharmacy Education in Saudi Arabia

**DOI:** 10.3390/healthcare12181806

**Published:** 2024-09-10

**Authors:** Mohammed M. Alsultan, Mohamed A. Baraka, Abdullah K. Alahmari, Mahmoud E. Elrggal, Mansour A. Mahmoud, Marwan A. Alrasheed, Shahad O. Alkahlah, Arjwan M. Alqarni, Manar M. Alghamdi, Abdulaziz H. Al Khzem, Bashayer M. Alshehail, Mansour M. Alotaibi

**Affiliations:** 1 Department of Pharmacy Practice, College of Clinical Pharmacy, Imam Abdulrahman Bin Faisal University, Dammam 34212, Saudi Arabia; 2210040082@iau.edu.sa (S.O.A.); 2190003018@iau.edu.sa (A.M.A.); 2190005111@iau.edu.sa (M.M.A.); bmalshehail@iau.edu.sa (B.M.A.); 2 Pharmacy Department, Fatima College of Health Sciences, Abu Dhabi P.O. Box 4589, United Arab Emirates; mohamed.baraka2020@gmail.com; 3 Clinical Pharmacy Department, Faculty of Pharmacy, Al-Azhar University, Cairo 3857, Egypt; 4 Department of Clinical Pharmacy, College of Pharmacy, Prince Sattam Bin Abdulaziz University, Al-Kharj 11942, Saudi Arabia; a.alahmari@psau.edu.sa; 5 Pharmacology & Toxicology Department, Faculty of Medicine, Umm Al-Qura University, Al Qunfudah 28821, Saudi Arabia; merggal@uqu.edu.sa; 6 Department of Pharmacy Practice, College of Pharmacy, Taibah University, Al-Madinah Al-Munawara 42353, Saudi Arabia; mammm.99@gmail.com; 7 Department of Clinical Pharmacy, College of Pharmacy, King Saud University, Riyadh 11451, Saudi Arabia; malrasheed1@ksu.edu.sa; 8 Department of Pharmaceutical Chemistry, College of Clinical Pharmacy, Imam Abdulrahman Bin Faisal University, Dammam 34212, Saudi Arabia; ahalkhzem@iau.edu.sa; 9 Pharmacy Practice Department, College of Clinical Pharmacy, King Faisal University, Alhofuf 31982, Saudi Arabia; mmqalotaibi@kfu.edu.sa

**Keywords:** telepharmacy, pharmacy students, telemedicine, pharmaceutical technology, education

## Abstract

Telepharmacy education should be incorporated into the curricula due to its beneficial effects on students, providing pharmacy services during their practice. Therefore, this study aims to explore the knowledge and perceptions of pharmacy students regarding the integration of telepharmacy services into their education curriculum in Saudi Arabia. A cross-sectional study was conducted using an online survey from 1 June to 30 September 2023, among pharmacy students from five universities in Saudi Arabia. The questionnaire was divided into three sections, and descriptive statistics and a generalized linear model were used for analysis. A total of 523 pharmacy students participated. Approximately half of the students were aware of telepharmacy, and only one-quarter had studied it as part of their curriculum. Students believed that telepharmacy education should cover communication, reimbursement, and training for virtual patient interactions. There was a significant positive correlation (*p* < 0.0001) between the knowledge and perception scores. In addition, students who had heard about telepharmacy before and those with a “somewhat” confidence level showed a significantly positive correlation with knowledge scores (*p* = 0.01). In conclusion, perception scores, students who had heard of telepharmacy, and those with a “somewhat” confidence level were all positively correlated with pharmacy students’ understanding of telepharmacy. This study underscores the importance of integrating telepharmacy education and practical training into pharmacy curricula to prepare future pharmacists for the evolving healthcare landscape.

## 1. Introduction

Patients can access telehealth services online to obtain affordable long-distance medical care easier than hospital-based care [1,2]. Telemedicine played a positive role during the coronavirus disease 2019 (COVID-19) pandemic, which provided faster and more affordable care to patients who needed regular hospital visits [3,4,5]. In addition, the use of MinuteClinic telehealth visits by patients has been associated with more convenience and patient satisfaction [6]. Therefore, the benefits of employing telemedicine services have been gaining favor with patients recently [7]. Telehealth visits increased dramatically at Oregon Health and Science University from 1100 prior to the COVID-19 pandemic to 13,000 during the pandemic [8]. There have also been reports of comparable increases in telehealth use in China, Brazil, France, and Switzerland [9,10].

These virtual consultation services were applied during the COVID-19 pandemic in the United States (US), the United Kingdom (UK), Canada, Australia, the United Arab Emirates (UAE), and China to provide patient counseling regarding therapy [11,12,13,14]. Thus, implementing telepharmacy services has an impact on increasing the role of pharmacists and interventions by approximately 42% due to the comfortable access of services and consultations [12]. In addition, a study of community pharmacists in Jordan found that 91% of them thought that telepharmacy was helpful to receive quicker medical feedback. Despite not being aware of telepharmacy, the majority of pharmacy students at the University of Tennessee and University of Jordan Health Science Centers said that they would be willing to use it and believed it could save time [15,16]. The telepharmacy services in Balkan countries have shown their beneficial effect on patients who needed optimal services to address their chronic illnesses during the COVID-19 pandemic, as provided by the pharmacist [17]. In addition, using remote pharmacy services in China to educate patients about their prescriptions had a positive impact during the pandemic [18]. In the USA, using clinicals for patients who needed online pharmaceutical consultation enhanced the role of pharmacists during the pandemic [19]. Effective services can be provided by communicating with patients using mobile health applications, short-message services from mobile phones, and online services [20,21]. As part of the Saudi 2030 Vision, the Saudi Ministry of Health is expanding patient counseling services by extending the use of technology in healthcare. Thus, the “SEHA-App” was launched to provide visual medical consultations, enabling users to communicate with medical professionals across all of Saudi Arabia to obtain guidance regarding their health and medications [22]. A study conducted in Saudi Arabia reported that pharmacists must obtain adequate training in telepharmacy to advance in their careers [23]. Therefore, the significance of technological services during the COVID-19 pandemic has been emphasized in earlier research, demonstrating its advantages for the hospital system.

This transformation in healthcare underscores the importance of integrating technology into pharmaceutical services. According to several research studies, hospital pharmacists have a high preparedness for telepharmacy, but pharmacy students have little understanding of the practice [24,25,26]. Thus, telepharmacy education should be incorporated into the curricula through simulations and experiential training due to its important and beneficial effects on students and their ability to provide pharmacy services during their practice [17,18,19]. The Saudi Arabian 2030 vision and the country’s high level of technological innovation highlight the importance of the telepharmacy services offered in the country. However, no study has examined pharmacy students’ knowledge and perceptions after the COVID-19 pandemic and the development of technological services in the Kingdom of Saudi Arabia. Therefore, this study aimed to investigate the knowledge and perceptions of pharmacy students regarding the integration of telepharmacy services into their education curriculum in Saudi Arabia.

## 2. Materials and Methods

### 2.1. Study Design, Settings, and Population

A cross-sectional study was conducted using an online survey from 1 June 2023 to 30 September 2023. This study was performed at the following five universities in the Kingdom of Saudi Arabia: Imam Abdulrahman Bin Faisal University, King Saud University, Prince Sattam Bin Abdulaziz University, Tabia University, and Umm Al-Qura University. Pharmacy students who had completed their preparatory year were eligible to participate in the survey. These five institutions were selected because they are old, have the largest student numbers, and are located in various parts of Saudi Arabia.

### 2.2. Study Tool, Sampling, and Sample Size

The questionnaire was established based on an extensive literature review of previously published studies [24,25,26]. To maintain content and face validity, we shared the survey with a five-pharmacy faculty for their feedback and comments. Pilot testing was conducted among 18 pharmacy students across all colleges. These students were excluded from the final analysis. The reliability coefficient of the survey was verified, and Cronbach’s alpha was 0.72 for the knowledge and perception sections, which is considered reliable. The questionnaire was divided into three sections, with 41 questions on demographic information, knowledge, and perceptions. Nine items were used to evaluate demographic variables, ten items were used to evaluate the students’ knowledge, and sixteen items were used to examine their perception regarding the implementation of telepharmacy service integration into their pharmacy education. Six additional questions were added for fifth year and internship students. To evaluate the students’ knowledge of telepharmacy in education, a scale with two responses (yes, and no) was used. One point was used for yes and zero was used for no. We calculated the total knowledge score for each student and then calculated the median for the total sample and used it to classify students’ knowledge into high knowledge >=7 or low knowledge <7. In addition, a 5-point Likert scale was used to assess students’ perceptions according to the following responses: strongly disagree = 1, disagree = 2, neutral = 3, agree = 4, and strongly agree = 5. The total perception score for each student was calculated; then, the median (56) was calculated for the total sample. Classification was either high >=56 and low <56. We used the convenience sampling technique in this study, and the survey was shared via the email address and study group platforms of each college. The estimated number of pharmacy students in Saudi Arabia is around 15,000 [27]. Using the online Raosoft^®^ sample size calculator, we looked for a 50% response in the surveyed items with a confidence interval of 95% and 5% margin error. Therefore, the minimum sample size for our study was equal to 375.

### 2.3. Statistical Analysis

We used the frequencies (percentages) and mean, along with their standard deviation (SD), to present the sociodemographic characteristics of our participants. The generalized linear regression model was applied to determine the factors that significantly affect the knowledge score. Therefore, the covariates that were added to the model were perception score, age, gender, pharmacy program, heard about telepharmacy before, and confidence. All statistical analyses were performed using the SAS 9.4 software. A *p*-value < 0.05 was considered statistically significant.

## 3. Results

### 3.1. Sociodemographic Characteristics of Participants

In total, 523 participants were included in this study. The mean (standard deviation) age of the study participants was 21.65 (±1.51) years (range: 18–27 years). Males and females were represented almost equally, with 261 males and 262 females. Most participants were Pharm. D program students, at 367 (70.2%), and 156 (29.8%) were B. Pharm program students. In total, 171 (32.7%) were from Imam Abdulrahman Bin Faisal University, 108 (20.7%) were from Prince Sattam Bin Abdulaziz University, 90 (17.2%) were from King Saud University, 86 (16.4%) were from Umm Al-Qura University, and 68 (13%) were from Taibah University (Table 1). In addition, only 281 (53.7%) students had heard the term “telepharmacy” before, whereas 139 (26.6%) students had studied telepharmacy as a course or chapter in their curriculum.

### 3.2. Knowledge of Pharmacy Students Regarding Telepharmacy

**Figure 1:**
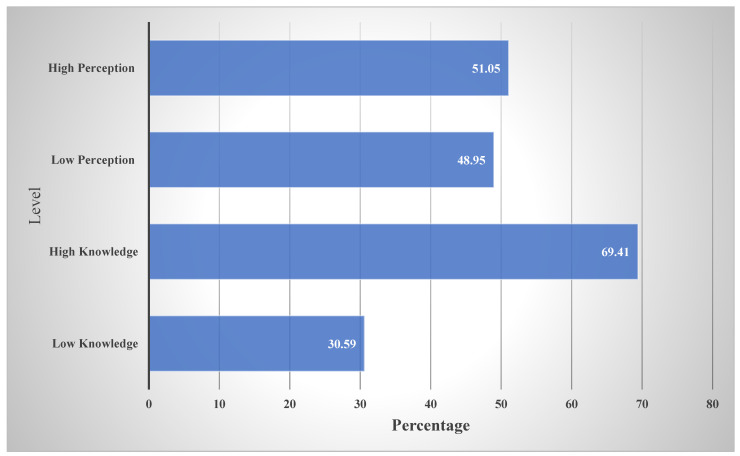
Knowledge and perception of telepharmacy among students.

Most study participants (363 [69.41%]) showed high levels of knowledge about telepharmacy, while only 160 (30.59%) showed low knowledge (Figure 1). Of the 523 students, 434 (82.9%) believed that telepharmacy is a practice in which pharmacists use telecommunication to manage pharmacies and patient care remotely; 429 (82%) believed telepharmacy is the provision of pharmaceutical care to patients residing at a distance through the use of telecommunication and information technologies; 397 (75.9%) believed that telepharmacy helps to reduce infection transmission by providing pharmaceutical care to mildly infected patients remotely; 350 (66.9%) believed that telepharmacy technology can be used virtually anywhere at any time; 364 (69.6%) believed that implementing telepharmacy requires special tools and space in the pharmacy to communicate with patients and healthcare providers; 120 (22.9%) believed that telepharmacy is not an essential service for pharmacists; 404 (77.2%) believed that telepharmacy education should include topics on the use of telecommunication technologies; 429 (82%) believed that telepharmacy education should include topics on communication between patients and service providers; 311 (59.4%) believed that telepharmacy education should include topics regarding payment for such services and the policies governing telepharmacy; and 395 (75.5%) believed that the preparation and training of patients and pharmacists for virtual meetings should be covered in telepharmacy education (Table 2).

### 3.3. Perception of Pharmacy Students toward Telepharmacy

Of note, 267 students (51.05%) had high perceptions of telepharmacy, whereas 256 (48.95%) had low perceptions (Figure 1). Additionally, 116 students (22.2%) believed they had sufficient knowledge of telepharmacy; 75 (14.3%) believed that only patients with infectious diseases require telepharmacy services; 283 (54.1%) believed that elderly patients receiving multiple medications (polypharmacy) were the most eligible for telepharmacy services; 330 (63.1%) believed that patients living in rural areas were the most eligible for telepharmacy services; 373 (71.4%) believed that it is crucial to include telepharmacy in undergraduate education in Saudi Arabia; 409 (78.2%) believed that it is crucial to provide telepharmacy training to pharmacists working in Saudi Arabia; 433 (82.8%) believed that the government could encourage telepharmacy implementation during and after the pandemic; 369 (70.5%) believed that implementing telepharmacy education will increase their ability to identify, prevent, and solve drug-related problems (DRPs); 418 (79.9%) believed that pharmacists’ involvement in telepharmacy will improve health awareness and the clinical outcomes of patients; 418 (79.9%) believed that pharmacists’ involvement in telepharmacy will improve the quality of health services, 375 (71.7%) believed that implementing telepharmacy education will improve the appreciation of patients toward the pharmacist’s role; 202 (38.6%) believed that the pharmacy curriculum is too overloaded to accommodate telepharmacy education; 254 (48.6%) believed that they feel more comfortable communicating with the patient face to face than via telepharmacy; 189 (36.2%) believed that in the future, telepharmacy will diminish the social and empathic aspects of care due to automation, decreasing its therapeutic value; 212 (40.5%) believed that telepharmacy services should only be provided by a clinical pharmacist; and 141 (26.9%) believed that pharmacists should focus on drug-dispensing services and leave the provision of telehealth services to physicians (Table 3). Among the study participants, 159 (30.4%) were fifth-year pharmacy students, of which 102 (64.2%) were interested in pursuing a career in telepharmacy in the future. Of note, 56 (35.2%) believed that they had acquired sufficient knowledge to allow them to provide telepharmacy services to their patients, 121 (76.1%) believed that not providing telepharmacy services during the pandemic could cause major health problems, 127 (79.9%) believed that pharmacy students should receive telepharmacy training during their training, 120 (75.4%) believed that telepharmacy implementation should be an essential part of pharmaceutical care provision, 63 (39.6%) felt confident about their ability to communicate effectively with patients via telepharmacy, 82 (51.6%) felt somewhat confident, and 14 (8.8%) did not feel confident.

### 3.4. Predictors of the Knowledge Score among Pharmacy Students

Pharmacy students’ knowledge of telepharmacy indicated a positive correlation with perception scores (*p* < 0.0001). In addition, students’ knowledge scores were significantly positively correlated with students who had heard about telepharmacy and those with a “somewhat” confidence level (*p* = 0.01) (Table 4).

## 4. Discussion

Our study explored the knowledge and perceptions of pharmacy students toward the integration of telepharmacy into their pharmacy curricula in Saudi Arabia. Most study participants were Pharm. D students. Almost half of the students were aware of the term telepharmacy; however, only one-quarter of them had studied telepharmacy as a chapter or course. Most students had a fair knowledge of telepharmacy. Most respondents believed that topics such as communication, the reimbursement of telepharmacy services, and training pharmacists for virtual patients should be part of telepharmacy studies. Around one-fifth of the students reported that they had sufficient knowledge of telepharmacy. When asked about the patient population eligible to receive telepharmacy services, the respondents reported that polypharmacy patients and those living in rural areas were the most likely to benefit from the provision of such services. Many study participants emphasized the importance of including telepharmacy in undergraduate courses while training current pharmacists on telepharmacy provision, and highlighted the importance of government support for such initiatives. Moreover, pharmacy students commonly believed that the identification, prevention, and resolution of DRPs could be improved by providing telepharmacy services. Improving health awareness, patient outcomes, and the quality of health services are additional benefits of accepting telepharmacy. Approximately half of the study participants reported that they favored face-to-face communication with patients, but they disagreed that telepharmacy would diminish the social aspects of face-to-face care and decrease its therapeutic value.

Several Saudi Arabian pharmacy students showed a high level of proficiency with telepharmacy, which is consistent with previous research in different countries, including the US (60.3%) and Malaysia (67%) [16,17,18,19,20,21,22,23,24,25,26]. Therefore, the recent adoption of digital healthcare services following COVID-19 may account for the robust knowledge of pharmacy students regarding telepharmacy in Saudi Arabia [28]. However, even with sufficient knowledge, students cannot always serve their patients effectively throughout their practice. We found that 60% of pharmacy students reported feeling unconfident or moderately confident when providing information. Therefore, sometimes the new practice of delivering information using online services rather than face to face can be challenging. To improve their confidence, all these topics should be included in the curriculum, as training or via simulations, before graduation [29,30]. The findings of this study, showing a predominantly high perception of telepharmacy among Saudi pharmacy students, are consistent with the global trends observed in similar studies. Elnaem et al. [26] reported that 61% of senior pharmacy students in Malaysia had a positive perception of telepharmacy, indicating the general acceptance of this emerging modality in pharmacy practice. This level of acceptance highlights the growing recognition of the potential of telepharmacy to improve healthcare delivery, especially in countries such as Saudi Arabia, where its integration into the curriculum is strongly advocated by students. Furthermore, the emphasis on the need for telepharmacy education, as seen in our study, is consistent with the findings of Patel [16], highlighting a global consensus on the importance of structured training to prepare future pharmacists for digital transformation in healthcare. However, concerns regarding the adequacy of telepharmacy training and its potential effect on the quality of patient interactions resonate across regions. The apprehension of students from Saudi Arabia regarding the loss of the social and empathetic aspects of telepharmacy was mirrored in a study by Skoy et al. [31], in which students were found to be more successful in face-to-face consultations than in those via telepharmacy. This indicates the need for a curriculum that balances technical proficiency and the development of the communication skills needed for remote healthcare delivery. Furthermore, despite the high readiness reported among Malaysian students [26], there remains a lack of comfort and proficiency in telepharmacy practice, indicating a universal challenge associated with the seamless integration of telepharmacy into pharmacy education and practice. The alignment of perceptions across different cultural and educational contexts further emphasizes the universality of these challenges and the global opportunity to improve pharmaceutical care through digital innovations such as telepharmacy.

In our study, there were strength and limitations; the major strength of this study is the extensive and diverse participation of pharmacy students across Saudi Arabia, providing a detailed view of their current perspectives and understanding of telepharmacy. This study revealed high perception levels and a strong knowledge base regarding telepharmacy among students, suggesting a solid foundation for the further development and integration of telepharmacy education into pharmacy curricula. These findings are consistent with the goals of Saudi Arabia’s Vision 2030, which emphasizes quality education and advanced healthcare systems. Nevertheless, the most significant limitation of this study is the geographic specificity of the study sample, which comprises solely Saudi Arabian pharmacy students, potentially limiting the generalizability of the findings to other regions or to different demographics, such as practicing pharmacists. Additionally, the dynamic nature of telepharmacy indicates that knowledge and perceptions are continually evolving. Therefore, our findings may become less relevant gradually. Another limitation is the reliance on self-reported data, which despite being a common methodology in surveys, can be subject to biases such as social desirability or inaccurate self-assessment.

These days, Saudi Arabia is implementing telepharmacy and other remotely accessible services as part of a digital transformation in healthcare that aligns with the Saudi Arabia 2030 goals. In addition, a range of health applications are available to patients, such as Tawakkalna, Sehhaty, and 937 Medical Tele-consultations, which offer improved accessibility and convenience in the provision of care [32,33]. Therefore, it is essential to prepare future pharmacists for the evolving healthcare landscape, where telepharmacy is becoming increasingly important. In addition, we recommend emphasizing the need for the strategic improvement and development of pharmacy curricula to address the gaps in knowledge and low perceptions of telepharmacy. The study also highlights the need for the continuous reassessment of student knowledge and perceptions, given the rapid changes in telepharmacy, to maintain current and effective educational content. Expanding future research to include various geographical areas and practicing pharmacists is recommended for gaining a broader understanding of global telepharmacy education. Additionally, integrating practical hands-on telepharmacy training into educational programs is important for improving student confidence and competence in this emerging field.

## 5. Conclusions

Based on our findings, the pharmacy students’ knowledge of telepharmacy was positively associated with their perception scores, whether they had heard about telepharmacy, and whether they had a “somewhat” confidence level. Improving telepharmacy education in Saudi Arabian pharmacy schools is required to better accomplish Saudi Vision 2030. This emphasizes how important it is for pharmacy courses to incorporate telepharmacy teaching in addition to the hands-on training that students should receive in order to be prepared before graduation. Therefore, this strategic refinement is crucial to ensuring that future pharmacists are thoroughly equipped to navigate the continuously evolving healthcare landscape.

## Figures and Tables

**Table 1:** Sociodemographic characteristics of participants.

Variable	Frequency	Percentage
**Sex**		
Female	262	50.1
Male	261	49.9
**Academic year**		
2nd year	72	13.8
3rd year	108	20.7
4th year	192	36.7
5th year	77	14.7
6th year (Internship)	74	14.1
**Pharmacy program**		
Bachelor of Pharmacy (B. Pharm)	156	29.8
Doctor of Pharmacy (Pharm. D)	367	70.2
**University**		
Imam Abdulrahman Bin Faisal University	171	32.7
King Saud University	90	17.2
Prince Sattam Bin Abdulaziz University	108	20.7
Taibah University	68	13.0
Umm Al-Qura University	86	16.4
**Have you heard of the term “telepharmacy” before?**		
Yes	281	53.7
No	242	46.3
**Have you studied telepharmacy as a course or a chapter in your curriculum?**		
Yes	139	26.6
No	384	73.4
**Have you received any training (as courses or internships) on how to use telepharmacy and telehealth to promote remote patient and healthcare-provider interactions? (*n* = 139)**		
Yes	30	21.6
No	109	78.4
**Have you received any simulation exercises for telepharmacy training in your college? (*n* = 139)**		
Yes	17	12.2
No	122	87.8

**Table 2:** Knowledge of telepharmacy among pharmacy students.

Question	No *N* (%)	Yes *N* (%)
I believe that telepharmacy is a practice in which pharmacists use telecommunication technology to manage pharmacies and patient care remotely.	89 (17.0)	434 (83.0)
I believe telepharmacy is the provision of pharmaceutical care to patients residing at a distance through the use of telecommunication and information technologies.	94 (18.0)	429 (82.0)
I believe that telepharmacy helps in reducing infection transmission by providing pharmaceutical care to mildly infected patients remotely.	126 (24.1)	397 (75.9)
I believe that telepharmacy technology can be used virtually anywhere at any time.	173 (33.1)	350 (66.9)
I believe that implementing telepharmacy requires special tools and space in the pharmacy to communicate with patients and healthcare providers.	159 (30.4)	364 (69.6)
I believe that telepharmacy is not an essential service for pharmacists.	403 (77.1)	120 (22.9)
Telepharmacy education should include topics on the use of telecommunication technologies.	119 (22.8)	404 (77.2)
Telepharmacy education should include topics on communication between patients and service providers.	94 (18.0)	429 (82.0)
Telepharmacy education should include topics on payment for such services and policies governing telepharmacy.	212 (40.5)	311 (59.5)
The preparation and training of patients and pharmacists for virtual meetings should be covered in telepharmacy education.	128 (24.5)	395 (75.5)

**Table 3:** Perception of pharmacy students toward telepharmacy.

Question	Strongly Disagree *N* (%)	Disagree *N* (%)	Neutral *N* (%)	Agree *N* (%)	Strongly Agree *N* (%)
I believe that I have sufficient knowledge about telepharmacy.	39 (7.5)	189 (36.1)	179 (34.2)	94 (18)	22 (4.2)
I believe that only patients with infectious diseases require telepharmacy services.	156 (29.8)	216 (41.3)	76 (14.5)	54 (10.3)	21 (4)
I believe that elderly patients receiving multiple medications (polypharmacy) are the most eligible for telepharmacy services.	30 (5.7)	71 (13.6)	139 (26.6)	197 (37.7)	86 (16.4)
I believe that patients living in rural areas are the most eligible for telepharmacy services.	15 (2.9)	37 (7.1)	141 (27)	209 (40)	121 (23.1)
I believe that it is important to include telepharmacy in undergraduate education in Saudi Arabia.	10 (1.9)	26 (5)	114 (21.8)	231 (44.2)	142 (27.2)
I believe that it is important to initiate telepharmacy training for pharmacists working in Saudi Arabia.	7 (1.3)	14 (2.7)	93 (17.8)	229 (43.8)	180 (34.4)
I believe that the government could encourage telepharmacy implementation during and after the pandemic.	7 (1.3)	5 (1)	78 (14.9)	221 (42.3)	212 (40.5)
I believe that implementing telepharmacy education will increase my ability to identify, prevent, and solve drug-related problems.	9 (1.7)	16 (3.1)	129 (24.7)	211 (40.3)	158 (30.2)
I believe that pharmacist’s involvement in telepharmacy will improve health awareness and the clinical outcomes of patients.	7 (1.3)	14 (2.7)	84 (16.1)	225 (43)	193 (36.9)
I believe that pharmacist’s involvement in telepharmacy will improve the quality of health services.	6 (1.1)	4 (0.8)	95 (18.2)	216 (41.3)	202 (38.6)
I believe that implementing telepharmacy education will improve patient’s appreciation toward the pharmacist’s role.	11 (2.1)	20 (3.8)	117 (22.4)	216 (41.3)	159 (30.4)
I believe that the pharmacy curriculum is too overloaded to accommodate telepharmacy education.	33 (6.3)	97 (18.5)	191 (36.5)	133 (25.4)	69 (13.2)
I believe that I feel more comfortable communicating with the patient face to face than through telepharmacy.	12 (2.3)	62 (11.9)	195 (37.3)	148 (28.3)	106 (20.3)
I believe that in the future, telepharmacy will diminish the social and empathic aspects of care due to automation, decreasing its therapeutic value.	41 (7.8)	103 (19.7)	190 (36.3)	130 (24.9)	59 (11.3)
Telepharmacy services should only be provided by a clinical pharmacist.	48 (9.2)	114 (21.8)	149 (28.5)	131 (25)	81 (15.5)
Pharmacists should focus on drug-dispensing services and leave the provision of telehealth services to physicians.	140 (26.8)	128 (24.5)	114 (21.8)	101 (19.3)	40 (7.6)

**Table 4:** Predictors of the knowledge score among pharmacy students.

Variables	Estimate	SE	*p*-Value *
**Intercept**	−1.81	3.44	0.60
**Perception score**	0.11	0.02	<0.0001 *
**Age**	0.01	0.145	0.95
**Male**	0.37	0.35	0.29
**Female** †			
**Pharm. D Program**	0.24	0.53	0.65
**B. Pharm Program** †			
**Heard about Telepharmacy before**	1.22	0.43	0.01 *
**Did not Heard about Telepharmacy before** †			
Somewhat confident	1.56	0.60	0.01 *
Very confident	0.93	0.61	0.13
Not confident†			

† Reference group, SE: standard error, * *p* ≤ 0.05.

## Data Availability

All the data of this project have been presented in this paper; however, the raw data are available upon request from the corresponding author (Mohammed M. Alsultan, M.M.A).

## References

[1] Gajarawala S.N., Pelkowski J.N. (2021). Telehealth benefits and barriers. J. Nurse Pract..

[2] Gardner M.R., Jenkins S.M., O’Neil D.A., Wood D.L., Spurrier B.R., Pruthi S. (2015). Perceptions of video-based appointments from the patient’s home: A patient survey. Telemed. e-Health.

[3] Mahtta D., Daher M., Lee M.T., Sayani S., Shishehbor M., Virani S.S. (2021). Promise and perils of telehealth in the current era. Curr. Cardiol. Rep..

[4] Powell R.E., Henstenburg J.M., Cooper G., Hollander J.E., Rising K.L. (2017). Patient perceptions of telehealth primary care video visits. Ann. Fam. Med..

[5] Sevean P., Dampier S., Spadoni M., Strickland S., Pilatzke S. (2009). Patients and families experiences with video telehealth in rural/remote communities in Northern Canada. J. Clin. Nurs..

[6] Polinski J.M., Barker T., Gagliano N., Sussman A., Brennan T.A., Shrank W.H. (2016). Patients’ satisfaction with and preference for telehealth visits. J. Gen. Intern. Med..

[7] Win A.Z. (2017). Telepharmacy: Time to pick up the line. Res. Soc. Adm. Pharm..

[8] Robinson E. (2020). OHSU Telehealth Rockets into ‘New Era of Medicine’: Global Pandemic Instigates Exponential Expansion of OHSU Telemedicine Program. OHSU News. https://news.ohsu.edu/2020/04/13/ohsu-telehealth-rockets-into-new-era-of-medicine.

[9] Koonin L.M., Hoots B., Tsang C.A., Leroy Z., Farris K., Jolly B.T., Antall P., McCabe B., Zelis C.B.R., Tong I. (2020). Trends in the use of telehealth during the emergence of the COVID-19 pandemic—United States, January–March 2020. MMWR Morb. Mortal. Wkly. Rep..

[10] Ohannessian R., Yaghobian S. (2020). The practicality of telemedicine and telehealth during the COVID-19 global outbreak. Eur. J. Public Health.

[11] Gross A.E., MacDougall C. (2020). Roles of the clinical pharmacist during the COVID-19 pandemic. J. Am. Coll. Clin. Pharm..

[12] Ibrahim O.M., Ibrahim R.M., ZAl Meslamani A., Al Mazrouei N. (2023). Role of telepharmacy in pharmacist counselling to coronavirus disease 2019 patients and medication dispensing errors. J. Telemed. Telecare.

[13] Hua X., Gu M., Zeng F., Hu H., Zhou T., Zhang Y., Shi C. (2020). Pharmacy administration and pharmaceutical care practice in a module hospital during the COVID-19 epidemic. J. Am. Pharm. Assoc..

[14] Unni E.J., Patel K., Beazer I.R., Hung M. (2021). Telepharmacy during COVID-19: A scoping review. Pharmacy.

[15] Muflih S.M., Al-Azzam S., Abuhammad S., Jaradat S.K., Karasneh R., Shawaqfeh M.S. (2021). Pharmacists’ experience, competence and perception of telepharmacy technology in response to COVID-19. Int. J. Clin. Pract..

[16] Patel K. (2021). Assessment of Knowledge, Attitude, Perception of Pharmacy. Applied Research Projects. https://dc.uthsc.edu/hiimappliedresearch/75?utm_source=dc.uthsc.edu%2Fhiimappliedresearch%2F75&utm_medium=PDF&utm_campaign=PDFCoverPages.

[17] Kovačević M., Ćulafić M., Vezmar Kovačević S., Borjanić S., Keleč B., Miljković B., Amidžić R. (2022). Telepharmacy service experience during the COVID-19 pandemic in the Republic of Srpska, Bosnia and Herzegovina. Health Soc. Care Community.

[18] Li H., Zheng S., Liu F., Liu W., Zhao R. (2021). Fighting against COVID-19: Innovative strategies for clinical pharmacists. Res. Soc. Adm. Pharm..

[19] Elson E.C., Oermann C., Duehlmeyer S., Bledsoe S. (2020). Use of telemedicine to provide clinical pharmacy services during the SARS-CoV-2 pandemic. Am. J. Health Syst. Pharm..

[20] Gul S., Ghaffar H., Mirza S., Fizza Tauqir S., Murad F., Ali Q., Zafar Malik A., Merrell R.C. (2008). Multitasking a telemedicine training unit in earthquake disaster response: Paraplegic rehabilitation assessment. Telemed. J. e-Health.

[21] Asseri A.A., Manna M.M., Yasin I.M., Moustafa M.M., Roubie F.M., El-Anssasy S.M., Baqawie S.K., Alsaeed M.A. (2020). Implementation and evaluation of telepharmacy during COVID-19 pandemic in an academic medical city in the Kingdom of Saudi Arabia: Paving the way for telepharmacy. World J. Adv. Res. Rev..

[22] Saudi MOH News All the Kingdom Will Be Covered by Telemedicine in Two Months 2018. https://www.moh.gov.sa/en/Ministry/MediaCenter/News/Pages/news-2018-03-06-006.aspx.

[23] Alanaz A., Albarrak A., Muawad R. (2021). 5PSQ-184 knowledge and attitude assessment of pharmacists toward telepharmacy in Riyadh City, Saudi Arabia. Eur. J. Hosp. Pharm..

[24] Muhammad K., Baraka M.A., Shah S.S., Butt M.H., Wali H., Saqlain M., Mallhi T.H., Hayat K., Fahelelbom K.M., Joseph R. (2022). Exploring the perception and readiness of pharmacists towards telepharmacy implementation; a cross sectional analysis. Peer. J..

[25] Tegegne M.D., Wubante S.M., Melaku M.S., Mengiste N.D., Fentahun A., Zemene W., Zeleke T., Walle A.D., Lakew G.T., Tareke Y.T. (2023). Tele-pharmacy perception, knowledge and associated factors among pharmacy students in northwest Ethiopia: An input for implementers. BMC Med. Educ..

[26] Elnaem M.H., Akkawi M.E., Al-Shami A.K., Elkalmi R. (2022). Telepharmacy knowledge, perceptions, and readiness among future Malaysian pharmacists amid the COVID-19 pandemic. Ind. J. Pharm. Educ. Res..

[27] Almaghaslah D., Alsayari A. (2021). Using a global systematic framework tool to identify pharmacy workforce development needs: A national case study on Saudi Arabia. Risk Manag. Healthc. Policy.

[28] Alkhalifah J.M., Seddiq W., Alshehri B.F., Alhaluli A.H., Alessa M.M., Alsulais N.M. (2022). The role of the COVID-19 pandemic in expediting digital health-care transformation: Saudi Arabia’s experience. Inform. Med. Unlocked.

[29] Peyre S.E., Peyre C.G., Sullivan M.E., Towfigh S. (2006). A surgical skills elective can improve student confidence prior to internship. J. Surg. Res..

[30] Wiliam D., Ainsworth L., Almeida L., Davies A., DuFour R., Gregg L., Guskey T., Reeves D. (2007). Content Then Process: Teacher Learning Communities in the Service of Formative Assessment. Ahead of the Curve: The Power of Assessment to Transform Teaching and Learning.

[31] Skoy E.T., Eukel H.N., Frenzel J.E., Schmitz T.M. (2015). Performance and perceptions. J. Pharm. Technol..

[32] Al-Kahtani N., Alrawiai S., Al-Zahrani B.M., Abumadini R.A., Aljaffary A., Hariri B., Alissa K., Alakrawi Z., Alumran A. (2022). Digital health transformation in Saudi Arabia: A cross-sectional analysis using Healthcare Information and Management Systems Society’digital health indicators. Digit. Health.

[33] Ministry of Health Sehha. https://www.moh.gov.sa/en/Support/Pages/MobileApp.aspx.

